# Structural, dielectric, and antimicrobial evaluation of PMMA/CeO_2_ for optoelectronic devices

**DOI:** 10.1038/s41598-024-52840-8

**Published:** 2024-01-31

**Authors:** Ahmed M. Bakr, Abdelfattah Darwish, A. A. Azab, Mohamed E. El Awady, Ahmed A. Hamed, Amir Elzwawy

**Affiliations:** 1 Spectroscopy Department, Physics Research Institute, National Research Centre (NRC), 33 El-Bohouth St., Dokki, Giza, 12622 Egypt; 2 Microwave Physics and Dielectrics Department, Physics Research Institute, National Research Centre (NRC), 33 El-Bohouth St., Dokki, Giza, 12622 Egypt; 3 Solid State Physics Department, Physics Research Institute, National Research Centre (NRC), 33 El-Bohouth St., Dokki, Giza, 12622 Egypt; 4 Microbial Biotechnology Department, National Research Centre (NRC), 33 El-Bohouth St., Dokki, Giza, 12622 Egypt; 5 Microbial Chemistry Department, National Research Centre (NRC), 33 El Bohouth St., Dokki, Giza, 12622 Egypt; 6 Ceramics Department, Advanced Materials Technology and Mineral Resources Research Institute, National Research Centre (NRC), 33 El Bohouth St., Dokki, Giza, 12622 Egypt

**Keywords:** Materials science, Nanoscience and technology, Physics

## Abstract

In the current report, we have successfully synthesized nanocomposites of PMMA incorporating different doping of CeO_2_ through a chemical approach. XRD results reflects decent matching for CeO_2_ nanoparticles with 29 nm crystallite size. FTIR spectroscopy demonstrates the characteristic functional groups validating the successful formation of the composite. The optical study of PMMA and the nanocomposites has proven that the optical properties such as band gap, refractive index, optical permittivity, and loss tangent factor are affected by adding CeO_2_ to the PMMA matrix.The peak residing around 420 nm by UV measurements is allocated to occurring electrons photoexcitation from the valence to conduction band inherent in CeO_2_. The dielectric measurements were achieved using broadband dielectric spectroscopy upon a wide span of frequencies (10^–1^–10^7^ Hz) and within temperatures from − 10 to 80 °C with a step of 10 °C. The permittivity decreases by adding CeO_2_ and the dielectric parameters are thermally enhanced, however, the temperature influence is based on CeO_2_ content, the higher the CeO_2_ amount, the higher the influence of temperature. The results of the nanocomposites revealed antibacterial activity counter to gram-positive bacteria strain (*S. aureus*, and *B. subtilis*), and gram-negative bacteria (*E. coli*, and *K. pneumoniae*), yeast (*C. albicans*, as well as fungi (*A. niger*). Inherently, the change in CeO_2_ concentration from 0.01 to 0.1 wt% delivers maximum influence against gram-negative bacteria. These PMMA CeO_2_-doped composites are beneficial for optoelectronic areas and devices.

## Introduction

The research is evolving rapidly to provide enhanced life conditions regarding the industrial and biomedical areas for numerous aspects. Nanotechnology and related nanoscale composites promote versatile characteristics that could not be accomplished for their counterpart coarse grains. The conjugation of polymers and inorganic 0D–3D nanoparticles in the nanocomposite structure offers a desirable set of physical and chemical merits based on their confinement size. Likewise, this conjugation paves the route for varied optimization in the optical, electrical, thermal, and biomedical specifications^1^.

Nanocomposites explicitly engineered for several biomedical applications are regularly stated as "biomedical nanocomposites". There exists a multitude of probable biomedical nanocomposites, which can be characterized into many interconnected classifications. The foremost biological practices for these biomedical nanocomposites encompass the delivery of drugs, wound dressings, antibacterial assets, tissue engineering, stem cell remedy, cancer treatment, cardiac prosthesis, peripheral organs, biosensors, artificial blood vessels, as well as enzyme immobilization^2^.

Polymethyl methacrylate (PMMA) is an amorphous polymer material that provides significant specifications due to its high transparency, increased mechanical properties, environmental safety, reduced cost, and feasible formation at limited temperatures accompanied by reduced thermal conductivity (around 0.0012 cal/s cm K)^3,4^. These merits nominate it as a prominent contender for the polymer matrix^1,5^. Furthermore, it is familiar with the ability to transmit light within around 300–1000 nm wavelength without significant loss which highlights its eminent optical performance^3,6^. PMMA is naturally insulating, however upon certain doping with the preceding metal oxides its electrical properties could be modulated^4^.

Metal oxides are a major abundant material on Earth^7^, composed of more than one element thus they might be modulated regarding their electrical, optical, and morphological specifications for a certain application. These metal oxides are familiar with their elevated bandgap value that delivers multiple properties beneficial for sensing appliances^8,9^, catalysis instruments^10^, storage devices, and optoelectronic devices^11,12^.

Cerium oxide nanoparticles, regularly known as nanoceria (CeNPs), are broadly recognized as highly auspicious metal oxide nanomaterials. The options available are either a pristine (bare) state or a state stabilized by ligands. Cerium-based nanoparticles (CeNPs) have grown in recognition as effective therapeutic agents in the fields of regenerative medicine and tissue engineering. In vitro studies have exploited their capability to promote cell proliferation, while in vivo experiments have exposed their role in expediting the healing process of lesions. These findings have significantly influenced the outlook on wound therapy, offering new possibilities for treatment^13–16^.

CeO_2_ nanoparticles are reported for their stability as well as biocompatibility where they demonstrate transparency within the visible light area and a 2.2 refractive index magnitude around 630 nm wavelength value^17,18^, besides a raised bandgap of 3.2 eV^19^. These advantages are favored for optoelectronic applications^20^. The CeO_2_ emerges as a significant metal oxide in the photocatalysis field^21^ Broker et al., studied the performance of photocatalytic activity for CeO_2_ when exposed to the sunlight directed towards the organic dye degradation^22^. Other reported research efforts revealed the impacts of the additives on CeO_2_ and their performance in catalytic^23^. TiO_2_ and CeO_2_ are usually combined for the collective features of their catalytic activity in general^24^. Former research works were directed to the impact of CeO_2_ on PMMA^1^ or TiO_2_/PMMA^25^ and CeO_2_/TiO_2_^26^.

The specifications provided by the polymeric network structure reinforced with a cerium oxide comprising elevated surface area, wide bandgap, conductivity, visible light transparency, provided electronic mobility, and antibacterial tendency offers a wide spectrum of photocatalysis, solar energy, and optoelectronic applications^18,27,28^.

In this report, we introduce the simple synthesis route to acquire PMMA/CeO_2_ with a limited doping molar ratio of the metal oxides to reveal their impact on the optical, electrical, and antimicrobial specifications. XRD, FTIR, optical, dielectric, and antimicrobial measurements were assessed via their conventical devices. This combined composite might shed light on the next stage of nanotechnological and industrial applications. The purpose of the intended effort is to improve the optical, electrical, and antimicrobial characteristics of the PMMA upon incorporating small portions of dopants. These specifications can be applied in a wide set of biomedical and optoelectronic applications.

## Experimental work

### Materials

Poly methyl methacrylate (PMMA), Sigma Aldrich, Toluene anhydrous (C_6_H_5_CH_3_), 99.8%, Merck, Cerium Oxide (CeO_2_), Sigma Aldrich.

### Preparation of PMMA/CeO_2_ nanocomposites

To prepare the samples, a certain amount of PMMA was dissolved in 50 ml of toluene, then CeO_2_ was added gradually. After that, they were homogenously blended by high-speed stirring for 2 h at 50 °C to acquire a consistent dispersion of the CeO_2_ in the PMMA matrix. Lastly, the constituted nanocomposite mixtures were decanted using petri dishes, and then they were aged for two weeks. The dry powders of PMMA and CeO_2_ were precisely weighted to acquire quantified weight percentages: zero, 0.01, 0.05, and 0.1 wt.%, which were marked as PMMA, PC1, PC2, and PC3, respectively.

### Measurements

The XRD measurements were conducted using Cu Kα-X-ray powder diffraction (XRD) by Bruker D-8 diffractometer instrument functioned at 35 kV and 30 mA, using a (0.05°) step size within a scanning range (5–70°). For measuring the IR spectra, we have employed the device Bruker optics VERTEX 7000 Fourier Transform Infrared Spectrometer. The acquired spectra were plotted in a spectral span of 4000–400 cm^−1^ having a 2 cm^−1^ resolution and 2 mm/s scanning speed. The optical measurements were attained by a Jasco V-570 spectrophotometer throughout the wavelength range (0.2–2.5 µm). The dielectric studies were achieved using broadband dielectric spectroscopy upon a wide range of frequencies (10^–1^ to 10^7^ Hz), and at temperatures between (− 10 to 80 °C) with a step of 10 °C. The technique employs an increased-resolution ALPHA analyzer having an active sample holder head (Model: Novocontrol, Montabaur, Germany), accompanied by an active sample head. A Quatro temperature controller system was utilized for the stabilization of temperatures less than 0.2 K, and pure nitrogen was used as a heating agent. Gold-plated stainless-steel electrodes with a 20 mm diameter having a configuration of parallel plate capacitor were used for the measuring process.

### Antimicrobial activity

To evaluate the CeO_2_ antibacterial efficiency. Test organisms comprised yeast (*Candida albicans* ATCC 10231), fungi (*Aspergillus niger* NRRLA-326), and gram-positive and gram-negative bacteria (*Staphylococcus aureus* NRRLB-767 and *Bacillus subtilis* ATCC 6633, *Escherichia coli* ATCC 25922, *Klebsiella pneumoniae* ATCC 10145) were employed. Flat polystyrene plates with 96 wells were functioned for performing the tests. Succeeding the addition of 10 µl of the test extracts (to obtain a final concentration of 500 g/ml) to 80 µl of lysogeny broth (LB broth), and 10 µl of the bacterial culture suspension media (log phase), further, the plates were incubated at 37 °C overnight. The absorbance amount was counted after around 20 h at OD600 in a Spectrostar Nano Microplate Reader (BMG LABTECH GmbH, Allmendgrun, Germany)^29,30^.

### Antibiofilm activity

The biofilm inhibitory efficiency of the pigment extract was assessed using the microtiter plate assay (MTP) approach. The experiment elaborated 96 wells of flat-bottom polystyrene plates, each encompassing two clinical pathogens (*E. coli* ATCC 25922 and *S. aureus* NRRLB-767). Every well was occupied by 180 μL of lysogeny broth (LB) and 10 μL of overnight bacterial growth sample. The plate was then incubated for 24 h at 37 °C with 10 μL of CeO_2_ at dissimilar concentrations, accompanied by a negative control entailing filtrate without the sample. To eradicate the presence of suspended bacteria, the liquid within each well was detached, and subsequently, each well was subjected to a washing procedure including the use of 200 μL of phosphate-buffered saline solution with a pH value of 7.2. The plate underwent a staining process for one hour, during which a solution of crystal violet with a concentration of 0.1% (w/v) was introduced to every well. Subsequently, rinsed by distilled water (200 μL), after which the plate was left to dry inside a laminar flow environment. The dry plate was treated with ethanol (95%), and the optical density (OD)-at 570 nm-was monitored using a SPECTROSTAR nano absorbance plate reader (BMG LABTECH) for quantification purposes^31,32^.

## Results and discussion

### XRD

**Figure 1:**
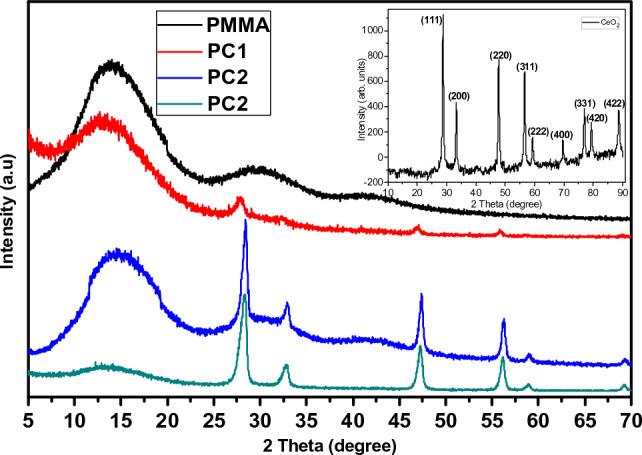
XRD pattern for PMMA, CeO_2_ nanoparticles, and PMMA/CeO_2_ nanocomposites. The right inset shows the indexed diffraction planes and their corresponding angles.

The PMMA/CeO_2_ synthesized nanocomposites constructed with CeO_2_ weight portions of 0, 0.01, 0.05, and 0.1 wt.%, were marked as PMMA, PC1, PC2, and PC3, respectively. The chemical structure was evaluated through XRD examination, as demonstrated in Fig. 1. Figure 1 (inset) illustrates the XRD spectrum of the CeO_2_ nanoparticles. The pattern displays that the distinctive peaks are found at 2θ = 28.9, 33.4, 47.7, 56.8, 59.1, 69.6, 77.1, 79.4, and 88.5° which are owing to (111), (200), (220), (311), (222), (400), (331), (420), and (422) lattice planes, correspondingly. The acquired lattice parameters of CeO_2_ nanoparticles coincide with former research data (JCPDS 34-0394)^33,34^. The XRD spectrum of cerium dioxide nanoparticles possesses wide peaks, which validate the construction of mini-sized nanoparticles. The familiar lattice parameters values attained from the XRD information were observed as (a = b = c = 5.411 Å), where (α = β = γ = 90°). The average crystallite dimension of CeO_2_ nanoparticles was identified using Scherrer’s equation^35–37^.

1 $$D=\frac{0.94\lambda }{\beta cos\theta }$$

Inherently, *λ* is incident X-ray source wavelength (1.54060 Å), *θ* is the Bragg’s diffraction angle, and *ß* is the full width at mid-maximum (FWHM) stated in radians^38^. The nominal crystallite size was calculated and found about 29 nm.

The PMMA sample displays three principal, wide XRD peaks, localized at 13.8° (the high-intensity band), besides two reduced-intensity peaks, noticed at 29.7° and 41.9°, which designate the amorphous character of the PMMA polymeric matrix^39^.

Additionally, this behavior was noticed in all the PMMA/CeO_2_ nanocomposites samples with low intensity. The PMMA/CeO_2_ nanocomposites samples' XRD diffraction patterns disclosed the amorphous nature of PMMA, signifying that CeO_2_ integration had no impact on the structural features of the PMMA and that no chemical interaction had occurred between the PMMA and CeO_2_.

### Optical analysis

**Figure 2:**
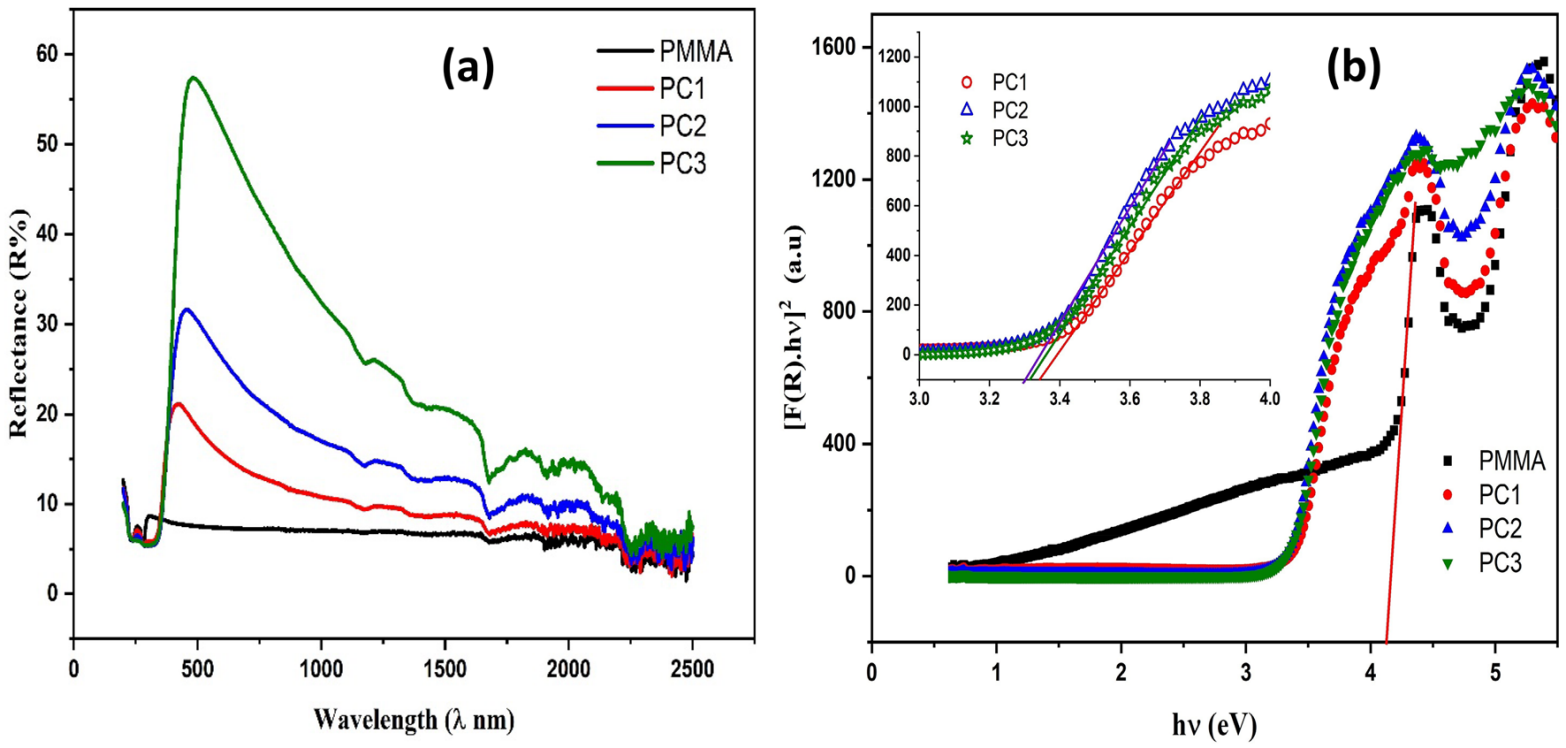
(**a**) Diffuse reflectance (R), (**b**) Tauc’s plot of PMMA and PMMA/CeO_2_ nanocomposites.

The investigation of the optical aspects of the prepared samples provides powerful details about their electronic properties and design. When examining materials using spectroscopy, the electronic vibration states are applicable. The electronic excitation is represented by the UV–Vis diffuse reflectance (DRS) part of the electromagnetic (EM) spectrum, and the energy levels are determined by the chemical bonds existent throughout the composite structure. To determine the optical properties of PMMA doped with CeO_2_ nanoparticles, the UV–Vis spectra were reported. Figure 2 reveals the UV spectra for all samples. The UV–Vis spectrum of PMMA displays very weak reflectance peaks at 300 nm, while the nanocomposites exhibit reflectance peaks and their intensity increases with CeO_2_ content in PMMA matrix. The peaks of nanocomposites were at 424, 456, and 480 nm for PC1, PC2, and PC3 respectively. Moreover, the reflectance of the nanocomposites is altered by different quantities of CeO_2_, resulting in a corresponding change in absorbance. The Kubelka–Munk relation was employed to measure the degree of change in the band gap energy (Eg),^40,41^

2 $$F\left(R\right)= \left(\frac{\alpha }{S}\right)= \left[\frac{{\left(1-R\right)}^{2}}{2R}\right]$$

F(R), α, and S are all present, and denoted as the Kubelka–Munk function, the absorption coefficient, and the scattering factor, correspondingly.

The optical energy bandgap (Eg) was evaluated by the proposed method introduced by Tauc equation^42^,

3 $$F\left(R\right)h\nu =A (h\nu -{E}_{g}{)}^{r}$$

where hv is the incident radiation's photon energy., A expresses a constant depending on the electronic transition probability, Eg denotes the optical band gap. The power factor, expressed by r, varies based on the type of transition. Additionally, the electronic transition's characteristics can be specified by defining the value of r. For direct transitions, r can be either 1/2 or 3/2, while for indirect transitions, r is equal to 2 or 3, depending on whether they are allowed or disallowed, respectively^43^. Normally there are two categories of materials for insulators and semiconductors: direct band gaps and indirect band gaps. The valence band maximum (VBM) and conduction band minimum (CBM) in direct band gap materials concur at the identical zero-crystal momentum position (i.e., wave vector *k* = 0)^44^. In this occasion, r receives the magnitude of 1/2. When the quantum selection rule prohibits a direct transition amongst the valence band maximum (VBM) and conduction band minimum (CBM) in certain materials, this transition is referred to as a forbidden direct transition. In this case, the value of r is equal to 3/2. An indirect electron transition arises when the VBM and the CBM are not located on the identical wave vector. In this instance, the VB to CB electronic transition with the appropriate crystal momentum magnitude will continually be associated with the absorption/emission of phonon energy^44^.

The optical energy gap is calculated from the relation (F(R)hν)^2^ versus hν through extrapolating the linear part of the curve to zero as illustrated in Fig. 2b. The band gap variation is shown in the inset figure, which is an amplification of Fig. 2b. The energy band gap of pure PMMA was determined to be 4.15 eV and it is consistent with numerous earlier reports^45,46^. The energy band gap of the nanocomposite samples was found 4.15, 3.34, 3.30, and 3.31 eV for pure PMMA, PC1, PC2, and PC3 respectively. Evidently, as the CeO_2_ content elevates in PMMA matrix, the optical energy band gap magnitudes drop. The addition of CeO_2_ nanoparticles in the polymeric host matrix leads to the emergence of extra absorption bands in the UV and visible portions of the optical spectra^47^. The decrease in the energy band gap could potentially be explicated by the intermolecular interactions that occur between the polymer and CeO_2_ particles. The gradual accumulation of CeO_2_ in the polymer matrix promotes significant modifications in the composites' structure, such as the enhancement of the conjugated chain lengths, resulting in a reduction of the band gap^47^. When assessing the electronic and optoelectronic qualities of a material, the composite's refractive index and optical dielectric characteristics are crucial. The refractive index of the PMMA/CeO_2_ nanocomposites can be considered using the following equation^48^,

4 $$\left\{n= \left[\frac{\left(1+R\right)}{\left(1-R\right)}\right]+\sqrt{\frac{4R}{{\left(1-R\right)}^{2}}-{k}^{2}}\right\}$$

**Figure 3:**
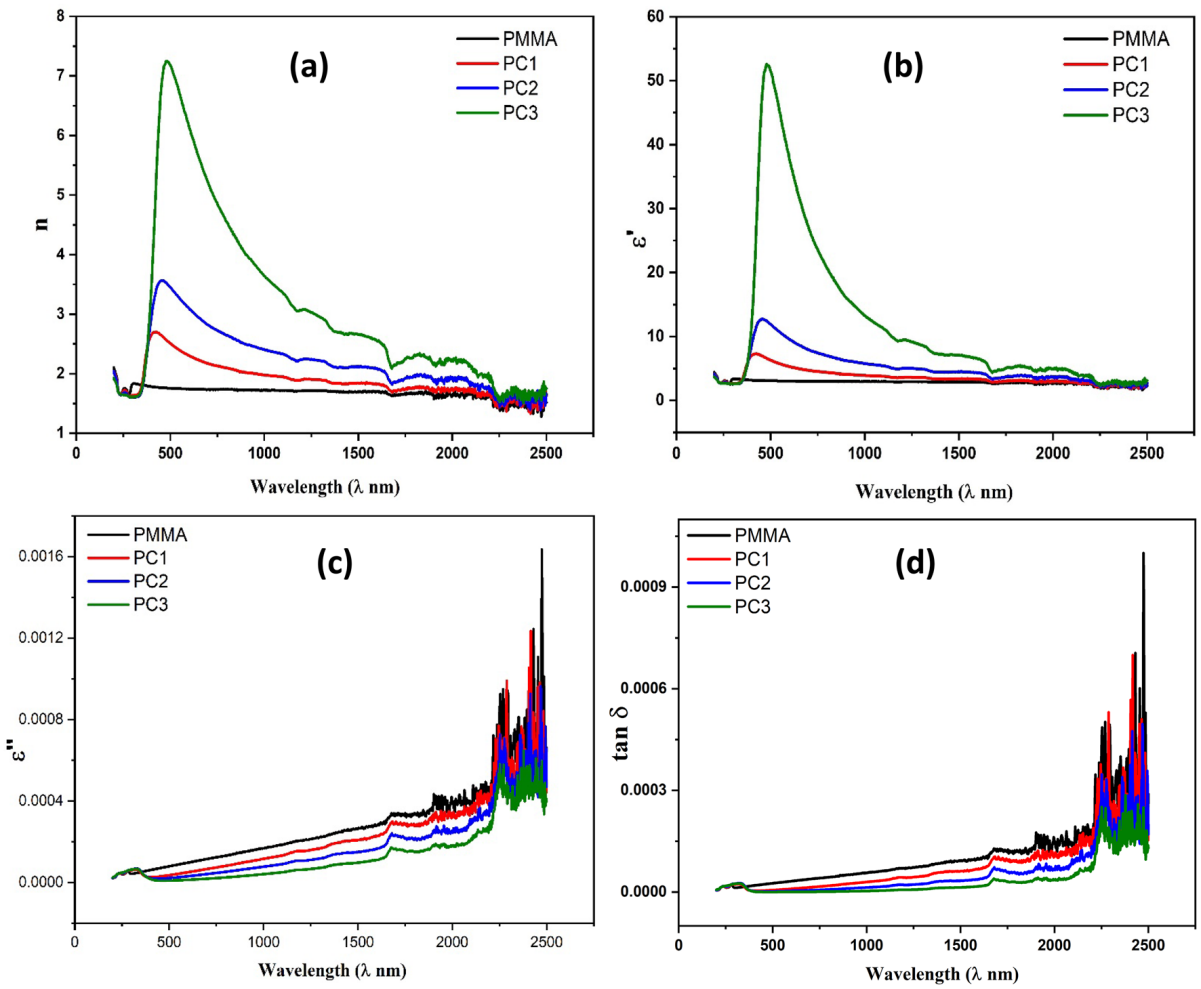
(**a**) The propagation of the refractive index (n), (**b**) Real section of the optical permittivity (εʹ), (**c**) Imaginary part of the optical permittivity (εʹʹ) and (**d**) Optical dielectric loss tangent (tanδ) of PMMA and PMMA/CeO_2_ nanocomposites.

Herein, k represents the extinction coefficient and has been determined via the relation (k = αλ/4π), where λ is the wavelength. Figure 3a demonstrates the relationship between the refractive index (n) and wavelength for both PMMA and the nanocomposites that were developed. The refractive index upsurges with raising the wavelength to reach the maximum value then decreases for all samples. Figure 3a shows that as CeO_2_ content enlarged, the refractive index of the nanocomposites also increased. The rise in the refractive index may be accredited to the change in PMMA structure due to CeO_2_ addition, which leads to the enhancement of the conjugated chain lengths.

The optical permittivity is connected with the refractive index (n), and extinction coefficient (*k*) via the following equation^48^,

5 $${\varepsilon }{\prime}+i{\varepsilon }^{{\prime}{\prime}}={({n}^{*} )}^{2}=(n+ik{)}^{2}= {n}^{2}- {k}^{2}+i 2nk$$

Figure 3b depicts the relationship between the real part of optical permittivity (εʹ) and wavelength. The real part of optical permittivity (εʹ) shows the same trend as the refractive index where it increases with frequency to a maximum value and decreases and increases with CeO_2_ content. The increased optical permittivity with CeO_2_ content might be ascribed to the generation of additional free charges triggered by the presence of CeO_2_, acting as a polarization center, hence enhancing the polarizability of the nanocomposites. The imaginary part of optical permittivity (εʹʹ) versus wavelength in Fig. 3c demonstrated the same behavior of the real part of optical permittivity with wavelength, but the behavior of the imaginary part of dielectric (εʹʹ) with CeO_2_ concentration is opposite to real part of optical permittivity (εʹ), which decreases with increasing CeO_2_ concentration, as illustrated in Fig. 3c. The loss factor (dielectric loss tangent) was either calculated through the subsequent relation^49^:

6 $$\left[{\text{tan}}\delta = \frac{{\varepsilon }^{{\prime}{\prime}}}{{\varepsilon }^{\prime}}\right]$$

The relationship ruling wavelength and optical dielectric loss, for pure PMMA and PMMA/CeO_2_ nanocomposites is displayed in Fig. 3d. The optical dielectric loss (tan δ) was found to decrease as the concentration of CeO_2_ in the nanocomposites increases and it increases with frequency.

### FTIR analysis

**Figure 4:**
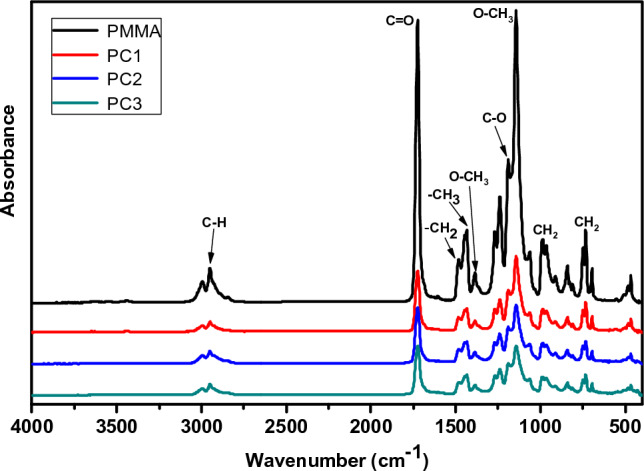
FTIR spectrum for PMMA, and PMMA/CeO_2_ nanocomposites.

(FTIR) spectroscopy is an advanced tool that might be employed to determine the handy functional groups of the prepared nanocomposite samples. As infrared radiation overrides via the sample, a specific ratio of the radiation intensity would be captivated and that will be revealed in the FITR spectrum. According to occurring stretching and bending vibrations within the samples, the IR radiation absorption appears at specific frequency values depending on the fabric of the sample. The chemical formula, sample’s functional groups can be identified by following the absorption bands or peaks in the wavenumber range 400 cm^−1^ to 4000 cm^−1^ of the FTIR spectrum. The FTIR spectrum of PMMA displays two peaks at 299 and 2950 cm^−1^, as presented in Fig. 4, which are assigned to C-H stretching vibration. The intense band positioned at 1724 cm^−1^ was attributed to C=O stretching vibration, whereas the two peaks that appeared at 1487 and 1442 cm^−1^ were assigned to –CH_2_ and –CH_3_ asymmetric stretching or deformation of PMMA. The peaks presented at 1383, 1203, and 1140 cm^−1^ are credited to O–CH_3_ deformation, C–O stretching, and –O–CH_3_ stretching of PMMA, respectively. CH_2_ twisting, wagging, and rocking vibrations of PMMA are located at 1195, 990, and 745 cm^−1^. The FTIR spectra for PMMA/CeO_2_ nanocomposites samples demonstrate the main characteristic peaks of PMMA as it denoted the spectra, except for the emergence of small peaks located at 430 cm^–1^, which is accredited to the metal–oxygen bond of cerium dioxide nanoparticles.

## Dielectric study

**Figure 5:**
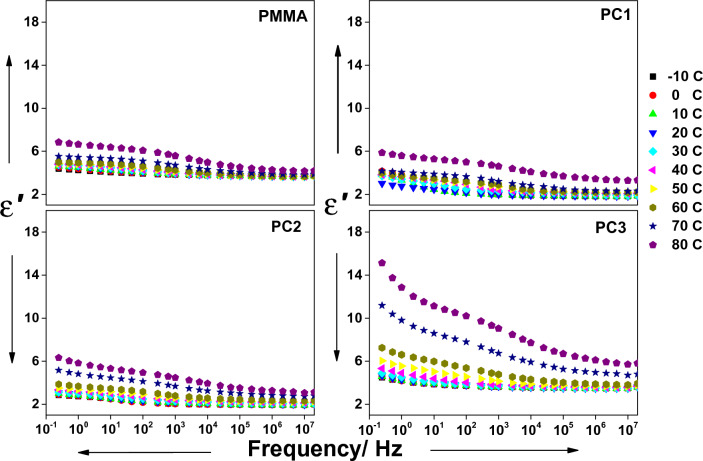
The real section of permittivity (ε′) vs frequency for all composites at temperatures between − 10 and 80 °C, as indicated.

**Figure 6:**
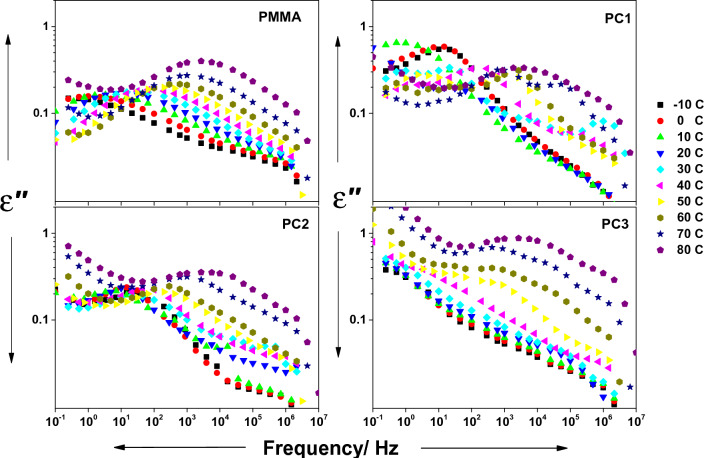
The imaginary part of permittivity (ε′′) vs frequency for the whole composites, at temperatures between − 10 and 80 °C, as indicated.

The permittivity is a complex function given by^50^

7 $${\varepsilon }^{*}={\varepsilon }^{\mathrm{^{\prime}}}-i{\varepsilon }^{\mathrm{^{\prime}}\mathrm{^{\prime}}}$$

where ${\upvarepsilon }^{\mathrm{^{\prime}}}$ is the real part which is a degree of the material's capability to store charges, and ${\upvarepsilon }^{\mathrm{^{\prime}}\mathrm{^{\prime}}}$ is the imaginary part which reflects the energy loss in a material. The variation of permittivity of all samples vs frequency at different temperatures is manifested in Figs. 5 and 6. The permittivity of PMMA has higher values at lower frequencies and then falls gradually with increasing frequency to be almost frequency-independent. The increased magnitude of permittivity in the low-frequency regime is believed to arise from the dipoles' alignment and obeying the applied field. The field variation becomes faster at a higher frequency and the dipoles lag and can't follow up the changes in the applied field which causes a drop in the polarization, hence the permittivity decreases and is no longer affected by frequency^50^. The addition of small amounts of CeO_2_ to PMMA altered its dielectric properties.

Adding CeO_2_ had two major effects. First, it hinders the polymeric chain mobility, which reduces its submission to the field and hence decreases the permittivity^51^. The second, it produces an interface inside the polymer matrix at which charges accumulate, which boosts the interfacial polarization and increases the permittivity. The permittivity is found to decrease with increasing CeO_2_ content, which means that the first factor prevails. The permittivity is thermally activated for all samples and the rate of increment of permittivity with temperature is dependent on CeO_2_ content, the highest content has the greatest rate. For the pure sample PMMA, by increasing temperature the permittivity values increase, the temperature increases the mobility of the dipoles and upsurges the conductivity, which increases the permittivity. By increasing the temperature for the samples containing small amounts of CeO_2_, besides the effects on PMMA dipoles, more charge carriers are liberated to accumulate at the interfaces between CeO_2_ and PMMA, which increases the permittivity.

The imaginary part of permittivity is presented in Fig. 6. The (ε″) of pure PMMA has three dielectric relaxations, interfacial polarization, conductivity contribution at low frequency, and β-relaxation at high frequency, which is ascribed to ester group with other accompanied methylene group^52^.

The conductivity contribution, which appeared in the curve as a linear decrease in the dielectric loss, is only noticed when the temperature is raised. The interfacial polarization and β-relaxation become faster with increasing temperature, and their corresponding peaks merge into a larger one. The conductivity contribution in the dielectric loss for samples PC1, PC2, and PC3 shows up at lower temperatures compared with the neat sample, and PC3 shows the highest conductivity contribution which suggests it is more conductive than other samples. The relaxation peaks are shifted to elevated frequencies with increasing CeO_2_ content which means their relaxation time decreases.

Electrical conductivity in the materials is a thermally activated procedure that occurs due to the motion of relatively loose charge carriers influenced by an electric field. The conductivity in numerous materials is frequency-dependent and obeys Jonscher's power law^53^,

8 $${\sigma }^{\prime}\left(\nu \right)={\sigma }_{dc}\left[1+{\left(\nu /{\nu }_{c}\right)}^{s}\right]$$

where ${\nu }_{c}$ the hopping frequency separating DC and AC regimes, ${\sigma }_{dc}$ represents DC conductivity and the exponent **s** represents a temperature-dependent parameter that holds $0<s\le 1$, depending on the conduction mechanism^54^.

**Figure 7:**
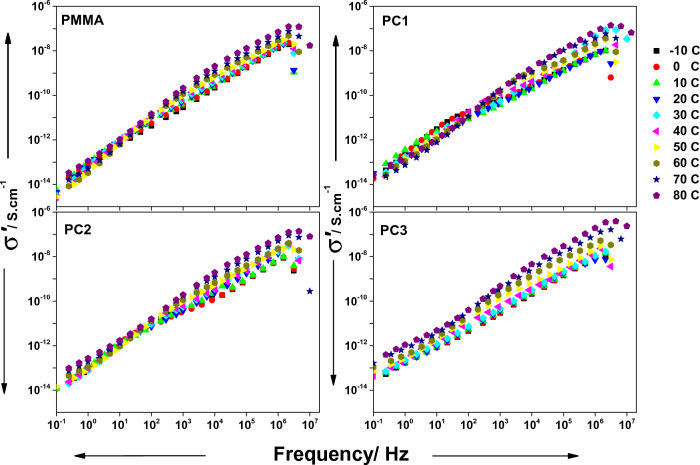
AC conductivity (σ′) vs frequency for all samples at temperatures ranging from − 10 to 80 °C, as indicated.

The AC conductivity vs frequency for all samples is shown in Fig. 7. The conductivity appears to be frequency dependent and no plateau or DC conductance is observed even when the temperature is raised or when CeO_2_ is added, which is the prime trait of insulators. The conductivity increases with frequency and it increases by increasing temperature or CeO_2_ content. The heat causes liberation to some charge carriers. The addition of CeO_2_ increases the crystallinity of the samples which provides facile routes for charge carriers’ transport. The sample PC3 has relatively higher conductivity and is slightly affected more than other samples by increasing temperature, which appears as semi-linear decrease in the dielectric loss at lower frequency.

### Antimicrobial and antibiofilm results

In our work, the antibacterial activity of CeO_2_ was evaluated using six distinct microorganisms (*B. subtilis* ATCC6633, *S. aureus* NRRLB-767, *K. pneumoniae* ATCC10145, *E. coli* ATCC25922, *C. albicans* ATCC 10231 and *A. niger* NRRLA-326). The outcomes in the documentation demonstrated antibacterial activity against the whole bacterial strains, mainly with increasing the concentration of CeO_2_ from 0.01 to 0.1, especially against gram-negative bacteria (*K. pneumoniae* ATCC10145 and *E. coli* ATCC25922). In contrast, the antifungal activity of the CeO_2_ against *C. albicans* ATCC 10231 and *A. niger* NRRLA-326 was insignificant. While the antibiofilm activity of CeO_2_ displayed a significant result for *S. aureus* NRRLB-767 with increasing the concentration of CeO_2_ (Tables 1, 2) The findings of this study align with those of former research^55^, which demonstrated the synthesis of a nanocomposite consisting of cerium oxide (CeO_2_) and graphene oxide. The nanocomposite exhibited promising antibacterial activity against various wound pathogens, including *E. coli, P. aeruginosa, S. aureus, and S. typhi*. The concentrations employed in the study were 25 μg/ml, 50 μg/ml, 75 μg/ml, and 100 μg/ml.

**Table 1:** Antimicrobial activity (%) of the PMMA/CeO_2_ nanocomposites.

		{Antimicrobial activity (%)}					
Compounds		Gram-positive		Gram-negative		Yeast	Fungi
		*(S. aureus) *NRRLB-767	*(B. subtilis) *ATCC 6633	*(E. coli) *ATCC 25922	*(K. pneumonia)* ATCC 10145	*(C. albicans) *ATCC 10231	*(A. niger) *NRRLA-326
PC 1	0.01 CeO_2_	13.08 ± 0.59	15.43 ± 0.59	22.39 ± 0.66	25.74 ± 0.49	0.0	0.0
PC 2	0.05	26.73 ± 0.58	28.54 ± 0.71	45.95 ± 0.30	49.60 ± 0.47	19.56 ± 0.68	17.62 ± 0.53
PC 3	0.1	38.85 ± 0.73	43.60 ± 0.89	59.46 ± 0.71	62.04 ± 0.51	34.83 ± 0.73	28.04 ± 0.48
Ciprofloxacin		96.01 ± 0.43	97.24 ± 0.18	98.07 ± 0.35	98.10 ± 0.27	–	–
Nystatine		–	–	–	–	97.16 ± 0.90	98.23 ± 0.16

**Table 2:** Biofilm inhibition ratio (%) of the PMMA/CeO_2_ nanocomposites.

Samples		Biofilm inhibition ratio (%)	
		*(E. coli)* ATCC 25922	*(S. aureus)* NRRLB-767
PC 1	0.01 CeO_2_	11.48 ± 0.73	17.40 ± 0.90
PC 2	0.05	22.06 ± 0.61	31.21 ± 0.47
PC 3	0.1	36.92 ± 0.80	42.75 ± 0.55

The membranes of both gram-positive and gram-negative bacteria were exposed to adsorption by the positively charged nanoparticles due to the electrostatic interaction, which can be credited to several mechanisms. The prolonged existence of nanoparticles (NPs) on the bacterial surface can be accredited to the electrostatic contact and the obstruction of the bacterial membrane, which suppress their penetration into the membrane. Subsequently, the introduction of nanoparticles (NPs) has the potential to vary the cellular membrane’s viscosity, hinder the functionality of particular ionic pumps, and ultimately disrupt the transport processes involved in the interchange of substances between the bacterial cell and its surrounding solution, thereby perturbing bacterial evolution^56^. After adsorption onto the exterior membrane of the bacterial cell, CeO_2_ has the potential to interact with and disrupt proteins. According to preceding studies^57^, the incidence of cerium ions has the potential to interrupt electron flow and respiratory processes in bacteria. Additionally, these ions can interact with thiol groups (–SH) or bind to transporters and/or porins, thereby impeding the delivery of vital nutrients. Further, the irregular forms and rough edges of CeO_2_ itself are responsible for causing physical impairment to bacterial membranes, principally in gram-positive bacteria case^58^.

## Conclusion

The feasible synthesis approach of PMMA/CeO_2_ nanocomposites is delivered in this work with varying amounts of CeO_2_. XRD crystallite size is moderately small as 29 nm. The investigation of the optical properties by UV–Vis showed that the band gap decreased from 4.15 eV for pure PMMA to 3.30 eV for PC2. The refractive index and optical dielectric constant increase with CeO_2_, while the imaginary part of the dielectric constant and optical dielectric loss tangent decrease with CeO_2_ concentration.The UV findings clarify electron photoexcitation at around 420 nm arising from the valence band and directing to the conduction band. The addition of small amounts of CeO_2_ to PMMA decreased the permittivity but it made the influence of temperature higher. The permittivity is thermally activated for all samples and the rate of increment of permittivity with temperature is dependent on CeO_2_ content. The dielectric spectra show three dielectric relaxations, interfacial polarization, conductivity contribution at low frequency, and β-relaxation at high frequency. The relaxation peaks are shifted to higher frequencies with increasing temperature or CeO_2_ content, which means their relaxation time decreases and they move faster. The conductivity is frequency dependent which is the prime trait of insulators and it increases by increasing temperature or CeO_2_ content. The antimicrobial specifications of the nanocomposites depict an antimicrobial activity towards gram-positive and gram-negative bacteria, further, yeast and fungi. These results might be beneficial and applied in the optoelectronic and biomedical application areas.

## Data Availability

The data related to or connected with the work are all encompassed in the manuscript and its supporting information.
